# Warburg效应及其对肿瘤转移的影响

**DOI:** 10.3779/j.issn.1009-3419.2015.03.09

**Published:** 2015-03-20

**Authors:** 慧君 魏, 丽丽 郭, 清华 周

**Affiliations:** 300052 天津，天津医科大学总医院，天津市肺癌研究所，天津市肺癌转移与肿瘤微环境重点实验室 Tianjin Key Laboratory of Lung Cancer Metastasis and Tumor Microenvironment, Tianjin Lung Cancer Institute, Tianjin Medical University General Hospital, Tianjin 300052, China

**Keywords:** Warburg效应, 机制, 肿瘤转移, Warburg effect, Mechanism, Tumor metastasis

## Abstract

肿瘤细胞葡萄糖代谢的一个特点就是在氧含量正常的情况下依然选择利用糖酵解，即Warburg效应。Warburg效应的内在机制十分复杂，可能与癌基因激活、抑癌基因失活，糖代谢酶表达异常和肿瘤微环境改变等有关，具体的机制还有待进一步研究。Warburg效应这种代谢重编程与肿瘤的发生发展有着密切联系，为肿瘤细胞提供生长优势，帮助其逃避凋亡，同时为肿瘤的转移创造合适的环境，促进肿瘤转移。本文概述了Warburg效应的发生机制及其对肿瘤转移的作用。

## Abstract (en)

Cancer cells exhibit altered glucose metabolism characterized by a preference for aerobic glycolysis even when the oxygen content is normal, a phenomenon termed "Warburg effect". However the definite molecular mechanisms of Warburg effect remains unclear, recent works indicated that it might be related to the abnormal activity of the oncogene and tumor suppressor genes, also the change of tumor microenvironment, the abnormal expression of glucose metabolic enzyme and so on. Warburg effect has a relationship with tumor progression and provide suitable conditions for tumor metastasis. This review will summarizes the mechanism of Warburg effect and its effect on tumor metastasis.

肿瘤是以细胞异常增殖为特点的一大类疾病，其种类繁多，具有不同的生物学行为和表现，其中恶性肿瘤对人类健康的危害最为严重。据相关资料显示在我国城市居民疾病死因居于第一位的便是恶性肿瘤。恶性肿瘤的主要特征之一是肿瘤转移。研究^[1]^显示虽然只有1%的原发肿瘤细胞可完成转移的全部过程，但却导致了超过90%的癌症患者的死亡。因此，转移是恶性肿瘤最后的，最具破坏性的阶段，也是导致癌症死亡的主要原因^[2]^，肿瘤侵袭转移是一个连续、渐进的多因素、多步骤动态的过程，大部分的研究也都集中于导致和影响转移的相关分子机制上。近几年肿瘤代谢成为了人们的关注热点，部分研究表明肿瘤为了逃避代谢压力，发生了转移，而这个过程中通常伴随了能量代谢的重编程。这种能量代谢的重编程会使得肿瘤细胞获得相关的能力来逃避正常的细胞凋亡程序，进行增殖和迁徙。

正常细胞体内，葡萄糖会维持一个平衡状态，在缺氧状态时，葡萄糖会转变为丙酮酸进而转变为乳酸，当氧含量正常时，丙酮酸会进入三羧酸循环（tricarboxylic acid cycle, TCA）循环。而肿瘤细胞即使在有氧情况下也不利用线粒体氧化磷酸化产能，转而利用有氧糖酵解，即瓦博格效应（Warburg effect）^[3]^。尽管Warburg效应的机制并未被完全阐明，但是人们已经研究证实了一些关键的调控机制与其密切相关，譬如线粒体氧化磷酸化损伤、糖代谢酶表达异常、癌基因激活、抑癌基因失活和肿瘤微环境改变等^[4]^。而且有研究^[4]^表明，这种代谢重编程过程与肿瘤转移有着密不可分的关系，因此本文将对Warburg效应及其与肿瘤转移的关系进行综述。

## 1 Warburg效应及其发生机制

正常细胞体内，葡萄糖会维持一个平衡状态，在缺氧状态时，葡萄糖会转变为丙酮酸进而转变为乳酸，当氧含量正常时，丙酮酸会进入TCA循环。而肿瘤细胞的一个普遍特点是即使在氧含量正常的情况下，葡萄糖摄取量和乳酸的积累量也会逐渐升高，利用糖酵解作为主要能量代谢的来源，获得更高的糖分解能力，使得葡萄糖转变为乳酸来产生ATP，这种现象我们称为Warburg效应^[3]^。

Warburg效应代表着肿瘤细胞对葡萄糖利用方式由氧化磷酸化到糖酵解的转变，现在被认为是肿瘤的一大特征。这种能量代谢的改变受复杂因素的调控，包括肿瘤微环境的压力和基因的改变等。一方面增强糖酵解作用，一方面抑制线粒体氧化磷酸化^[4]^。Warburg内在机制十分复杂，尚未完全阐明，近年研究提出并证实了相应的一些调控机制。

### 1.1 癌基因和抑癌基因异常

肿瘤细胞糖酵解活性受癌基因和抑癌基因的调控。癌基因有促进细胞糖酵解和削弱线粒体氧化磷酸化功能的作用，抑癌基因可以抑制肿瘤的发生发展。癌基因的活化及过度表达和抑癌基因的失活可以异常调控糖酵解中的关键酶及重要蛋白分子，是致使肿瘤发生的主要原因。

如原癌基因*Ras*、*Src*基因突变激活后，变异的Ras^[5]^、Src^[6]^蛋白可以增加糖转运载体的表达，增加糖摄取和糖酵解，不受上游信号控制，持续促进细胞增殖；AKT1通过增加葡萄糖转运体4的表达，激活己糖激酶，刺激葡萄糖的摄取^[7]^；Myc的激活诱导糖酵解酶如乳酸脱氢酶（lactate dehydrogenase, LDH-A）和丙酮酸脱氢酶激酶（pyruvate dehydrogenase, PDK1）的表达上调从而诱导糖酵解^[8, 9]^。关于抑癌基因，现在越来越多的研究显示，它们的失活是肿瘤发生侵袭生长的主要原因。nm23、p53在肿瘤转移过程中已被确认有着较为重要的作用。*nm*23基因的低表达已经在多种高转移性肿瘤中被证实，其表达异常可影响微管聚合，导致染色体畸变和非整倍体形成从而驱动肿瘤转移，也可通过影响细胞骨架构成或G蛋白介导的细胞信号转导通路参与肿瘤的发生^[10]^；*p53*是一种重要的转录因子，也是肿瘤细胞中最常发生突变的抑癌基因，在调节线粒体呼吸和与糖酵解方式之间的平衡中发挥着重要作用^[11]^，他的突变可促进糖酵解和降低有氧呼吸，促使肝脏线粒体耗氧量下降，乳酸明显增多。p53通过调节相关靶基因表达，例如TIGAR等来控制糖酵解与线粒体氧化磷酸化之间的平衡^[12]^。除此之外，近年另一个抑癌基因*LKB1*也成为了人们的关注热点，他与AMPK形成的路径被证明与肿瘤的发展转移有着紧密联系。

### 1.2 糖代谢中相关酶

代谢中一些关键酶在Warburg效应中起到了重要的作用，关键酶的代谢改变可导致糖酵解能力增强，促进肿瘤细胞葡萄糖的摄取，增加了乳酸的积累量，从而进一步支持肿瘤的生长发展。如己糖激酶-Ⅱ（hexokinase-Ⅱ, HK-Ⅱ)、磷酸果糖激酶（phosphofructokinase, PFK）、丙酮酸激酶、乳酸脱氢酶、磷酸甘油脱氢酶等等。其中关键酶的一个共同特点就是都转变为胚胎亚型来支持增殖所需要的大规模代谢、合成^[13]^，为癌细胞提供了各种益处，譬如阻止细胞凋亡，增加侵袭性，对致癌作用有直接的影响。

糖异生途径中一个限速酶——果糖1, 6二磷酸酯酶催化F-1, 6-BP转化为F-6-P。有研究^[14]^表明在基底细胞样乳腺癌（basal-like breast carcinoma, BLBC）细胞中，人果糖-1, 6-二磷酸酶可以减少乳酸的产生，增加氧消耗，阻止糖酵解，增加氧化磷酸化，而FBP1的丢失可诱导糖酵解，导致葡萄糖摄入量的增高；还可以通过抑制线粒体复合物Ⅰ活性来激活氧化产物从而抑制氧消耗。在控制糖酵解过程中，FBP1是snail的一个主要下游靶点，在乳腺癌中利用snail复合物可以抑制FBP1，沉默钙粘着蛋白（E-cadherin）^[15]^，这样的一个代谢重编程导致了肿瘤细胞中肿瘤干细胞（cancer stem cell, CSC）类似特性的增加和维持，促进肿瘤发生和转移，在EMT和BLBC中是一个关键的致癌事件^[14]^。

有氧糖酵解是肿瘤细胞优先选择的代谢方式，活跃的糖酵解代谢有益于恶性肿瘤的增殖生存，增强癌细胞的侵袭能力^[16]^。

## 2 Waburg效应对肿瘤转移的作用

### 2.1 Warburg效应为肿瘤细胞提供生长增殖优势

癌细胞不同于正常细胞利用线粒体氧化磷酸化产能，它们大部分采取有氧糖酵解这种代谢方式，即Warburg效应。有氧糖酵解并不是有效的产能途径，每分子葡萄糖经糖酵解生成乳酸只产生2分子ATP，而经TCA循环可产生36分子的ATP，但是对于快速增殖的肿瘤细胞而言，有氧糖酵解是更有利的代谢选择^[16]^。肿瘤细胞受局部缺氧等微环境的影响，线粒体氧化磷酸化受到抑制，糖酵解可以补充产能，并且呈现较高的ATP/ADP比率^[17]^，而且有氧糖酵解满足了快速增殖的癌细胞对于大分子合成代谢的需要，提供了大量的代谢前体物质，其产生的大量碳水化合物代谢产物都分流进了各种生物合成途径中。例如糖酵解中间产物6-磷酸葡萄糖、6-磷酸果糖、磷酸甘油醛-3-磷酸转变成了5-磷酸核糖，合成核酸；磷酸烯醇式丙酮酸及丙酮酸可以合成氨基酸；G-6-P可以通过戊糖磷酸途径产生NADPH合成脂肪酸等，这都为肿瘤细胞提供了生长优势^[3]^。提高了肿瘤细胞的增殖生存能力，也是肿瘤细胞能够发生侵袭转移的基础。

### 2.2 Warburg效应帮助肿瘤细胞逃避失巢凋亡

失巢凋亡，一种特殊的细胞程序死亡，是由于细胞与细胞外基质或相邻细胞脱离接触而诱发的。失巢凋亡作为一种特殊的程序化细胞死亡形式，在机体发育、组织自身平衡、疾病发生中起重要作用，是肿瘤转移的一道障碍，也就是说拒绝失巢凋亡对癌细胞的转移扩散很重要^[18]^。

肿瘤细胞中对于调节细胞失巢凋亡敏感性来说，葡萄糖代谢是一个决定性的因素，有氧糖酵解减少了葡萄糖的氧化代谢，使得癌细胞对失巢凋亡的抵抗性增加，机制^[16]^在于有氧糖酵解中，PDKs、LDH等酶高表达，PDH等低表达，可以为癌细胞提供失巢凋亡阻力，帮助脱离细胞基质的癌细胞逃避失巢凋亡，延长他们的生存^[19]^，有研究表明PDKs是不依赖贴壁细胞存活的重要调节基因，也是癌细胞转移传播的一个调节基因；其次有氧糖酵解可以使癌细胞避免产生过多活性氧（reactive oxygen species, ROS），保持在一个约化态，克服失巢凋亡。所以有氧糖酵解可以促使更多的葡萄糖流向PPP途径，产生还原产物NADPH，作为细胞内主要的抗氧化剂保护细胞免受ROS影响，而且通过降低线粒体氧化代谢，避开ROS过表达，提高抗氧能力，保持氧化还原动态平衡，促进失巢凋亡的抵抗和肿瘤的转移。

### 2.3 Warburg效应下的肿瘤微环境对转移的影响

#### 2.3.1 重要的中间产物——乳酸

乳酸是糖酵解的最终产物，也是一种微环境因子。在肿瘤细胞中，葡萄糖经有氧糖酵解生成乳酸，其积累量不断增加造成了微环境的酸化。在实体瘤中乳酸的累积是恶性肿瘤发展中一个关键的早期事件，肿瘤乳酸代谢与癌侵袭性密切相关，肿瘤的乳酸量与远距离转移的发生率有正面联系，利用其可以预测肿瘤转移及患者的存活率^[13]^。研究^[20]^发现乳酸可以促进细胞迁移和聚集，促进免疫逃逸，诱导肿瘤转移。具体的机制在于：肿瘤细胞通过乳酸诱导的分泌物血管内皮生成因子（vascular endothelial growth factor, VEGF）来为增殖确保充足的氧和营养物，导致新血管生成，为细胞迁移创造环境，导致迁移增多。当肿瘤细胞释放大量乳酸到细胞外时，由于细胞内外乳酸浓度比率的变化，免疫细胞不能释放自身乳酸出去，从而最终因为自身乳酸窒息而亡，导致免疫逃避，从而促进肿瘤转移^[21, 22]^。肿瘤细胞借助单羧基转运体（monocarboxylate transporters, MCTs）分泌乳酸，Vegran等^[23]^证实乳酸可以通过MCT1、MCT4进入内皮细胞并转运，刺激NF-κB/白细胞介素8通路引起细胞迁移和血管形成，而且伴随着H^+^的运输，细胞内pH下降，酸性环境导致细胞内毒性T细胞的功能也会下降。一系列的反应促进肿瘤细胞的免疫逃逸，为癌细胞营造更适合的环境来进行转移侵袭，逃避了代谢压力从而避免凋亡。乳酸的积累是肿瘤微环境改变的一个重要方面，是肿瘤细胞转移的一个决定性事件。

#### 2.3.2 缺氧诱导因子

快速生长的肿瘤细胞中，局部供氧量不足，通过上调糖酵解来代替氧化磷酸化不足来补偿能量不足。缺氧诱导因子（hypoxia-inducible factor, HIF-1α）是迄今为止发现的唯一特异性缺氧状况下发挥活性的转录因子，是细胞适应低氧状态的关键分子^[19]^，他的活化可以诱导糖酵解酶及葡萄糖转运载体基因的表达，并且通过转录活化PDK，细胞色素C氧化酶COX4-2亚型来抑制线粒体氧化代谢，从而上调肿瘤细胞的有氧糖酵解^[24]^。而葡萄糖消耗增加，氧化磷酸化减少及乳酸的生成积累又可以诱导HIF1α表达。例如在有氧糖酵解中，乳酸的积累造成的酸性缺氧环境，及Warburg激酶AKT^[25]^的活化都会促进HIF1α的转录和翻译。HIF1α通过信号传导促进肿瘤细胞增殖、启动肿瘤血管新生和躲避细胞凋亡程序等来促进肿瘤转移——在肿瘤细胞缺氧条件下，调节多种代谢靶基因的高表达，提高肿瘤细胞增殖活性；HIF1α对血管生成起着中枢调节作用，HIF的表达可增强*VEGF*基因表达，增强VEGF的转录活性，而VEGF作为诱导肿瘤细胞血管生成能力最强的细胞因子^[26]^，在HIF1α促进下使得肿瘤新生血管增加从而提高肿瘤侵袭转移能力；肿瘤缺氧微环境中HIF1α高表达，存在了大量免疫抑制活性的细胞因子和生长因子促进了肿瘤细胞的免疫逃逸，而免疫细胞不但不能起到抗肿瘤的作用，反而促进了肿瘤细胞的侵袭和转移，加速了肿瘤的演进。

### 2.4 Warburg效应可能诱导上皮间质转化促进肿瘤转移

上皮间质转化（epithelial mesenchymal transition, EMT）是具有极性的上皮细胞转换成为具有活动能力的间质细胞并获得侵袭和迁移能力的过程，在肿瘤细胞侵袭过程中起着重要作用，与肿瘤细胞的侵袭和转移关系密切，是许多肿瘤侵袭转移的早期标志^[27]^。

Warburg效应对EMT的诱导可能涉及到了多种信号通路及分子机制，例如其中抑癌基因*LKB1*，转录因子snail等可能起到了一定作用。*LKB1*是一个已知的肿瘤抑制基因，在大多数癌症患者中都发生了由于体细胞突变而导致的*LKB1*基因的丢失，这种突变广泛存在于众多类型的恶性肿瘤中，如肺癌、结肠癌、乳腺癌等，在非小细胞肺癌中突变率更高达15%-35%^[28]^。有研究^[29]^表明LKB1可调节*E*-*cadherin*的表达从而调控EMT，E-cadherin作为EMT的一种重要标记物受到很多转录因子调控，其中研究较多的snail家族可识别并与E-cadherin基因启动子部位的E-box序列相结合，抑制其表达，近年一些研究证实，snail在一些恶性肿瘤中呈高表达，被认为是一个反映恶性肿瘤预后不良的重要生物学标记物。LKB1也许可以通过调控snail来对由Warburg诱导的EMT进行调节，从而调控肿瘤转移，但是具体机制还有待研究。其他关于Warburg与EMT关联的研究中，有实验^[30]^显示Warburg效应中一些相关酶，譬如柠檬酸盐等的突变丢失可能促进EMT发生，推进肿瘤恶性发展。

## 3 展望

肿瘤细胞能量代谢较正常细胞发生了异常，是一个复杂的过程，尤其糖代谢过程虽然已经有了大量研究但是其中各关键点依然还有很多疑问亟待解决，他对肿瘤转移的作用逐渐成为研究的热点。肿瘤中的Warburg效应表现出肿瘤细胞的高糖酵解速率，不仅提供了能量还提供了用于细胞生物合成的大量中间产物，为肿瘤快速增殖提供了合适的能量和营养，保证了肿瘤细胞的快速生长，帮助其逃避了代谢压力，譬如氧化损伤等，同时促进了肿瘤细胞的免疫逃逸，增加了他们的侵袭能力，为转移提供了合适的环境和条件。所以研究Warburg效应中关键的调控点有助于揭示肿瘤细胞转移的机制，为肿瘤的靶向性治疗提供很好的方向和策略。
